# Design of bionic active–passive hybrid-driven prosthesis based on gait analysis and simulation of compound control method

**DOI:** 10.1186/s12938-021-00962-9

**Published:** 2021-12-17

**Authors:** Xinsheng Xu, Xiaoli Xu, Ying Liu, Kai Zhong, Haowei Zhang

**Affiliations:** School of Medical Instrument and Food Engineering, University of Shanghai for Science and Technology, 516 JunGong Road, Shanghai, 200093 People’s Republic of China

**Keywords:** Active and passive hybrid drive, Two degrees of freedom ankle joint, Co-simulation, Compound P/PI feedback control

## Abstract

**Purpose:**

The purpose of this paper is to design a prosthetic limb that is close to the motion characteristics of the normal human ankle joint.

**Methods:**

In this study, combined with gait experiments, based on a dynamic ankle joint prosthesis, an active–passive hybrid-driven prosthesis was designed. On this basis, a real-time control algorithm based on the feedforward compensation angle outer loop is proposed. To test the effectiveness of the control method, a multi-body dynamic model and a controller model of the prosthesis were established, and a co-simulation study was carried out.

**Results:**

A real-time control algorithm based on the feedforward compensation angle outer loop can effectively realize the gait angle curve measured in the gait test, and the error is less than the threshold. The co-simulation result and the test result have a high close rate, which reflects the real-time nature of the control algorithm. The use of parallel springs can improve the energy efficiency of the prosthetic system.

**Conclusions:**

Based on the motion characteristics of human ankle joint prostheses, this research has completed an effective and feasible design of active and passive ankle joint prostheses. The use of control algorithms improves the controllability of the active and passive ankle joint prostheses.

## Background

In recent years, due to the continuous advancement of science and technology and the extension of the average life expectancy, countries have begun to develop into an aging society. At the same time, the proportion of people suffering from diseases has also increased due to the increase of the elderly people. Among them, the proportion of amputations due to blood circulation disorders is increasing. Up to now, more than 70% of amputations are caused by blood circulation disorders, such as arteriosclerosis and diabetes complications [1]. According to statistical research, as of 2020, the total number of diabetes patients in China has reached 129.8 million, and the overall prevalence of diabetes represent a growing trend. Patients due to diabetic complications caused by lower limb amputation, orthopedic amputations in all patients accounted for 50% [2]. In addition, the number of traumatic disabilities caused by external factors such as traffic accidents and natural disasters is also increasing year by year. It is estimated that by 2030, China will have 2.33 million lower limb amputations [3]. In traumatic amputation, the proportion of patients with lower limb amputation exceeds 90%, and most of them lose the ability to walk after the operation, which causes a lot of inconvenience to their daily life. The installation of prosthesis not only replaces the incomplete limbs in appearance, but also can effectively help patients, who suffer from lower limb amputations, to recover a certain amount of exercise ability and self-care ability, so that they can improve their quality of life. Whether it is a thigh amputation or a calf amputation, it is necessary to install an ankle joint prosthesis [4].

The passive ankle joint prosthesis has simple structure and low cost, which is a practical means to restore the walking ability of amputees. At present, most ankle joint prostheses on the market are passive, such as Ossur^©^LPVariflex®, Ottobock^©^Meridium® etc. This kind of prosthesis has a common disadvantage, that is, it cannot directly generate mechanical power, and the ankle joint muscles provide direct propulsion during the kick-off period. Amputees need to consume more energy when wearing this ankle joint prosthesis, so they cannot adapt to complex road conditions, such as ramps and stairs [5].

**Fig. 1:**
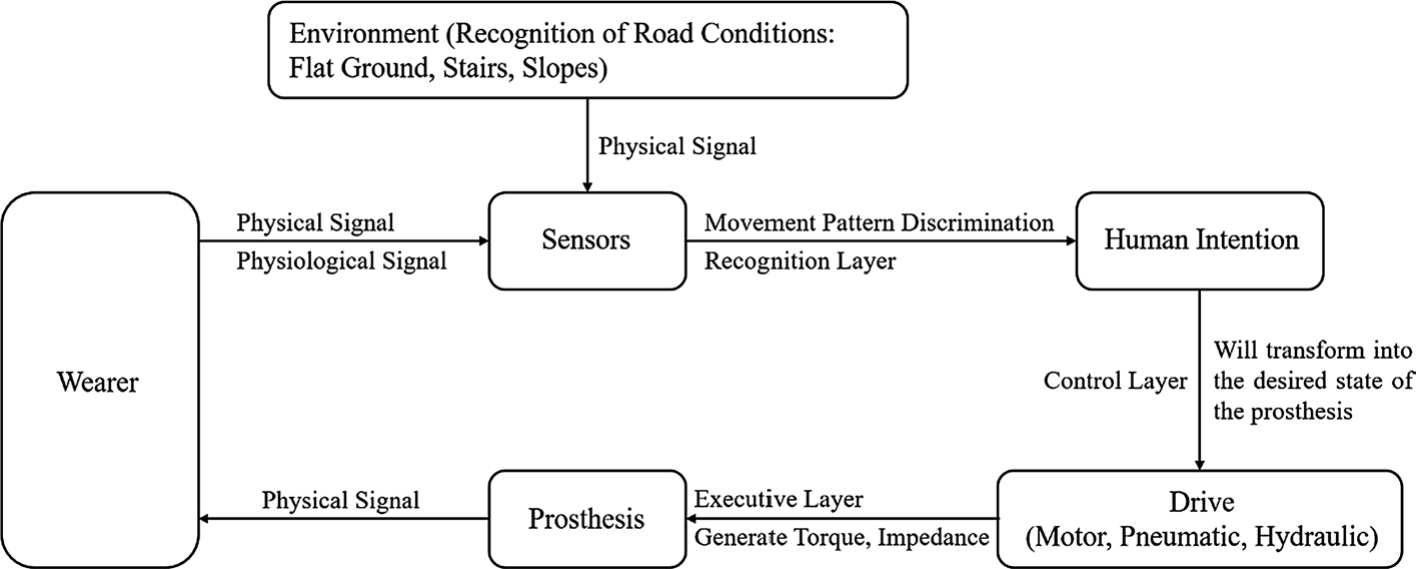
Control block diagram of powered lower limb prosthesis

Therefore, a dynamic ankle joint prosthesis that can provide active driving force came into being, which not only meets the daily needs of users (walking at medium and high speeds, climbing stairs, running, and jumping), but also ensures the stability of the legs during the support period and the flexibility of the ankle during the swing period. It can adjust the output torque to adapt to different sports and joint angle changes. In the active lower limb prosthesis, the recognition layer uses sensor data to determine the human movement pattern, the control layer maps the human body's intention to the control algorithm, and the executive layer generates torque to drive the prosthetic movement [4], the control block diagram of the powered lower limb prosthesis is shown in Fig. 1.

However, in terms of mechanism design, the main issues are the portability of the power supply, the specifications of the actuator and the bionic structure. In terms of control, it is mainly the optimization and improvement of strategies and algorithms [6, 7].

Therefore, this article aims to promote an active and passive ankle prosthesis suitable for people with lower limb amputation. And then design a real-time control algorithm based on compound P/PI feedback control. (Position Proportional and Speed Proportional Integral Regulator: According to the given value and the actual output value, the control deviation is formed. The proportional and integral of the deviation are linearly combined to form the control quantity, then the object is controlled.) Finally, the co-simulation is used to apply the control system to the virtual prototype model, so that the prosthesis has the gait motion characteristics close to the human body.

## Results

### Creation of the simulation model

**Fig. 2:**
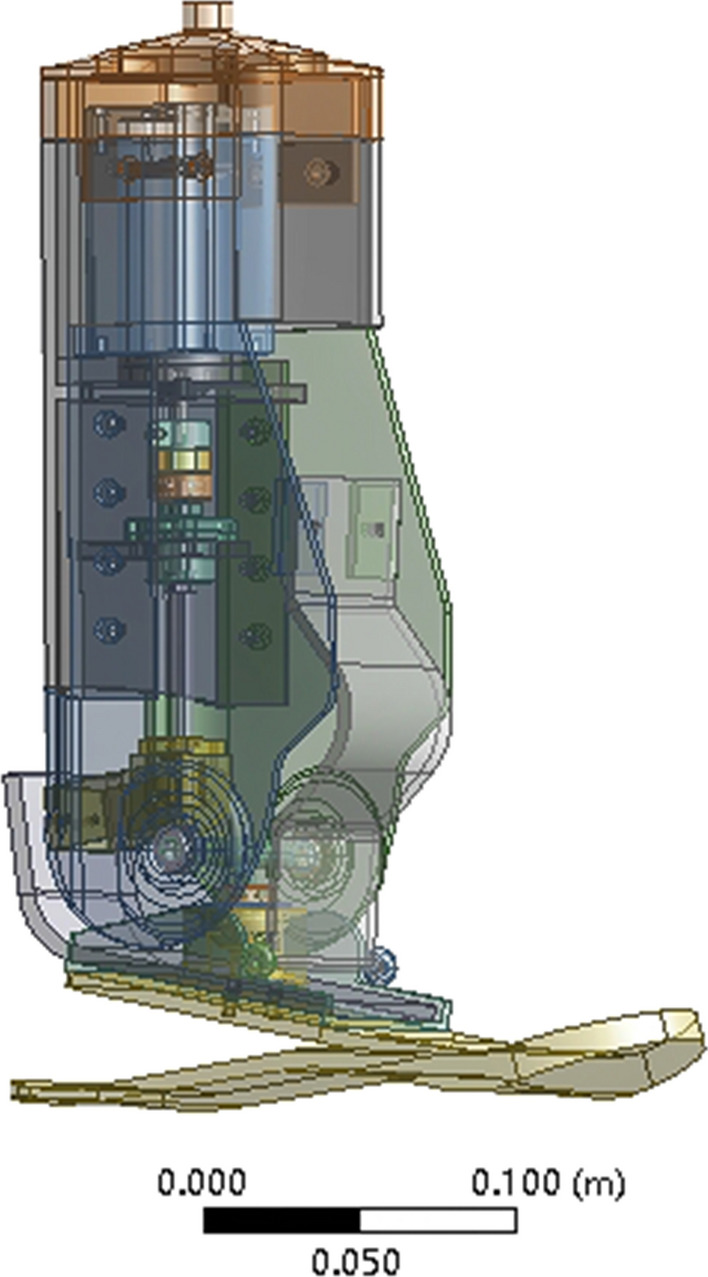
Mechanical structure of ankle joint prosthesis

According to the parameters of the ankle joint prosthesis, a three-dimensional model of each component is established through Catia. The mechanical structure of the assembled prosthesis is shown in Fig. 2. The overall height of the prosthesis is 353.66 mm, the maximum length is 248.167 mm, the maximum width is 74 mm, and the overall weight is 2.274 mm.

### Control algorithm optimization

**Fig. 3:**
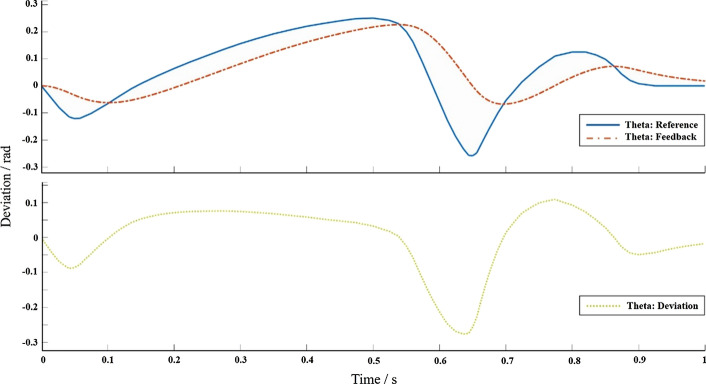
The simulation results of Simulink, the upper figure is the angle simulation curve, and the lower figure is the deviation curve

To test the effectiveness of the proposed control algorithm, a multi-body dynamic model and a P/PI control system simulation model were established in the NX-Simulink simulation environment, and co-simulation research was carried out. To realize the real-time interaction of information between the multi-body dynamics model and the control system model, the speed of the motor in the multi-body dynamics model is used as the output control quantity of the control system model, and a closed-loop control simulation environment between the prosthesis model and the Simulink control model is constructed. Set up the control system block diagram in Simulink, connect the control system with the Plantout module obtained in NX, and run the co-simulation. When Simulink and NX are running co-simulation, they run synchronously according to the sampling frequency set in NX, and the running conditions of the simulation can be observed in the two software, respectively (Fig. 3).

Servo stiffness refers to the ability of the servo control system to resist external interference to produce positional deviation. If the servo stiffness of the drive is insufficient, the servo system is prone to vibration, which seriously affects the control accuracy. Among them, the servo stiffness can be expressed by the following formula:

$$K_{g} = \left| {\frac{{T_{r} }}{\Delta X}} \right|$$

In the formula, $$T_{r}$$ is the external load torque, and $$\Delta X$$ is the position deviation of the servo system under the action of the external load torque. When the motor is selected, the motor armature resistance $$R_{a} = 0.4{\Omega }$$, inductance $$L_{a} = 10.6\;{\text{mH}}$$, and torque constant $$T_{n} = 60.3 \,{\raise0.7ex\hbox{${{\text{mN}} \cdot {\text{m}}}$} \!\mathord{\left/ {\vphantom {{{\text{mN}} \cdot {\text{m}}} {\text{A}}}}\right.\kern-\nulldelimiterspace} \!\lower0.7ex\hbox{${\text{A}}$}}$$ remain unchanged. Then the relationship between other parameters and servo stiffness needs to be determined. Set the interference as a step input signal, the servo stiffness value $$K_{g}$$ can be obtained by the formula, and the value of $$\Delta X$$ in the formula is the maximum value of the position deviation obtained by the simulation under the action of the interference torque.

After successful operation, the angle control effect is shown in Fig. 3, and the quantitative factors affecting the control system are shown in Table 1. It can be found from the table that the position loop proportional gain $K_{v}$, the speed loop proportional gain $K_{p}$, and the speed loop integral response time constant $T_{n}$ have a greater impact on the stability of the control system. For the speed loop filter time constant $T_{g}$, the greater the $T_{g}$, the less the servo stiffness, the increase in the number of oscillations before the system reaches the steady state. When the current loop proportional gain $K_{pi}$ increases to a certain extent, the servo stiffness of the system hardly changes. Therefore, increasing the position loop proportional gain $K_{v}$, increasing the speed loop proportional gain $K_{p}$, and reducing the speed loop integral response time constant $T_{n}$ can improve the servo stiffness and reduce the impact on system stability.

**Table 1:** Initial value and increase of control parameters

Control Parameter	${K}_{v}$	${K}_{p}$	${K}_{pi}$	${T}_{n}$	${T}_{g}$
Initial value	10	10,000	20	6	0.3
Change range	300%	300%	500%	250%	250%
Servo stiffness change range	120.2%	319.5%	122.2%	70.1%	80.5%

**Fig. 4:**
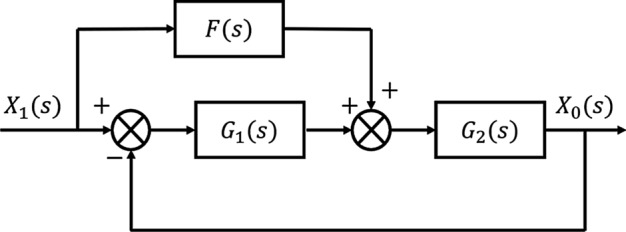
Compound control structure principle

However, after adjusting the quantification of each factor, it is found that the actual feedback value still has a large deviation from the demand value. For a control system, it should have a certain steady-state accuracy and good dynamic performance. The control system should accurately track the given input changes without being affected by external disturbances [10, 11]. Therefore, this paper further introduces the concept of compound control to reduce control deviation. The compound control is controlled according to the principle of invariance, the error is mainly caused by the input signal, so input compensation is generally selected for the input signal. The principle is shown in Fig. 4.

In Fig. 4, $G_{1} \left( s \right)$ is the system feedback control, $G_{2} \left( s \right)$ is the control object, $F\left( s \right)$ is the feedforward control, then the closed-loop transfer function of the system is

$$G\left( s \right) = \frac{{X_{0} \left( s \right)}}{{X_{i} \left( s \right)}} = \frac{{F\left( s \right)G_{2} \left( s \right) + G_{1} \left( s \right)G_{2} \left( s \right)}}{{1 + G_{1} \left( s \right)G_{2} \left( s \right)}}.$$

The system error transfer function is

$$G_{e} \left( s \right) = \frac{E\left( s \right)}{{X_{i} \left( s \right)}} = \frac{{1 - F\left( s \right)G_{2} \left( s \right)}}{{1 + G_{1} \left( s \right)G_{2} \left( s \right)}}.$$

When $F\left( s \right) = \frac{1}{{G_{2} \left( s \right)}}$, $\frac{{X_{0} \left( s \right)}}{{X_{i} \left( s \right)}} = 1$, that is, the output signal of the system can fully reproduce the given input signal. Then the steady-state and dynamic errors are zero, that is, the system has achieved complete invariance to the input. Therefore, $F\left( s \right) = \frac{1}{{G_{2} \left( s \right)}}$ is the condition of complete invariance of the input.

**Fig. 5:**
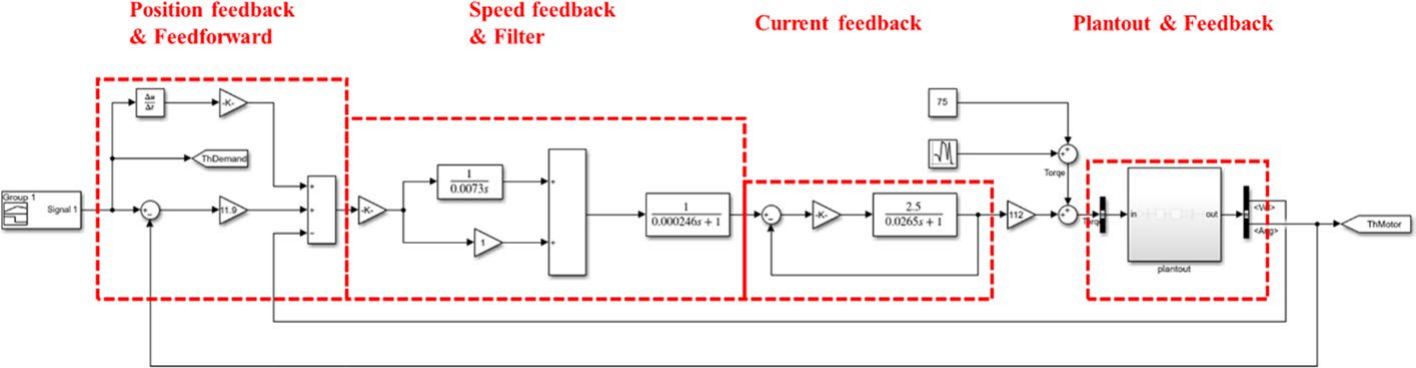
Compound P/PI control system for active and passive ankle prosthesis

**Fig. 6:**
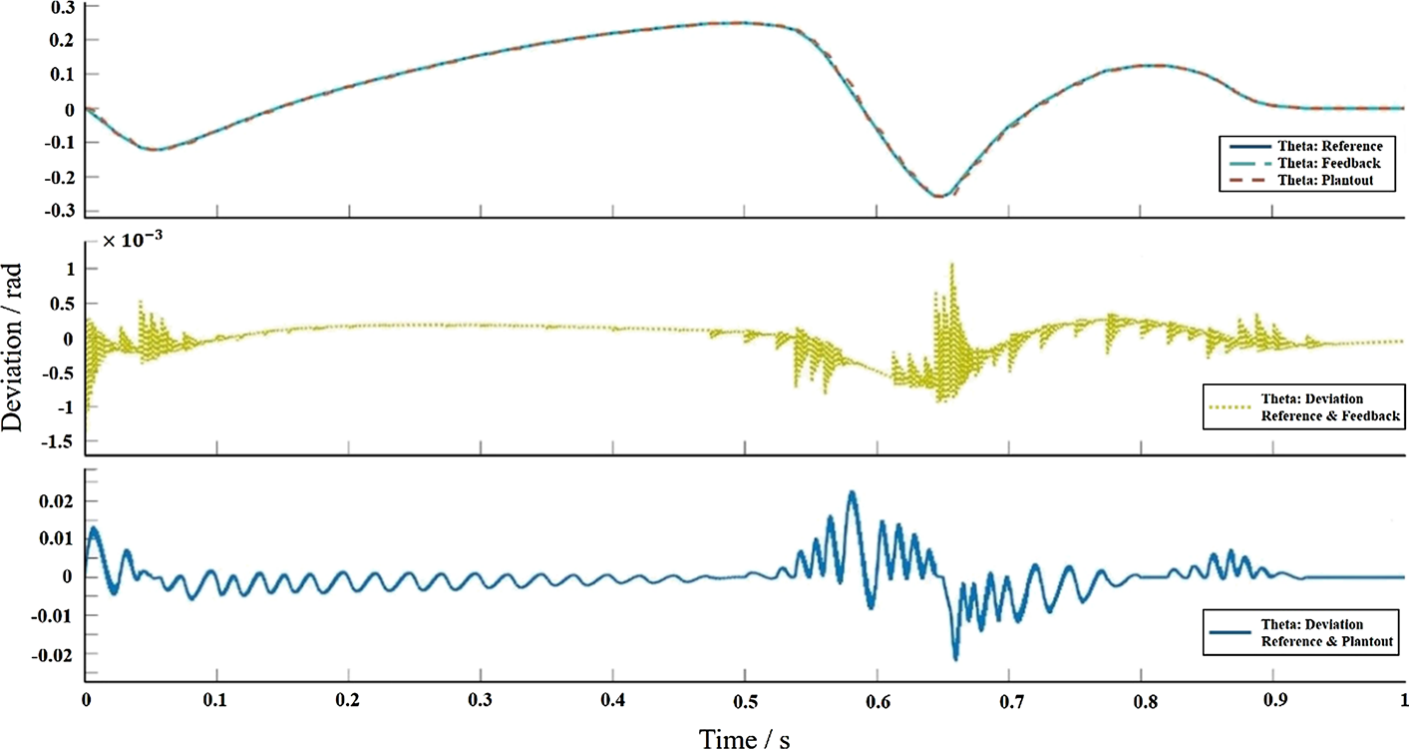
Comparison of the results of angle simulation

The updated desired angle signal can be converted into the change of the motor speed through the force servo control algorithm with feedforward compensation. Finally, the power source motor in the prosthetic multi-body dynamics model is output to realize the control of the model. Use Simulink to verify the feasibility of active and passive ankle joint prosthesis as a compound P/PI control system, the simulation system is shown in Fig. 5. Use Signal Builder to construct the ideal angle curve in the figure as the input signal of the compound control system. Differentiate the input signal to obtain the speed feedforward given signal. Therefore, when the angle curve measured by the experiment is used as the system input, the maximum error is $$- 1.5 \times 10^{ - 3} \sim 1 \times 10^{ - 3} rad$$, which is significantly higher than that of the control structure without feedforward. With the Plantout module, the maximum error is $$- 2.5 \times 10^{ - 2} \sim 2.5 \times 10^{ - 2} rad$$, the control accuracy is within the controllable range (The current control accuracy achieved in this paper satisfies: the curve does not fluctuate, remains smooth, and the error is between $$\pm 1^\circ / \pm 1.7 \times 10^{ - 2}$$). The parameter values of the compound P/PI control system are: $$K_{v} = 1.9{\raise0.7ex\hbox{${\text{m}}$} \!\mathord{\left/ {\vphantom {{\text{m}} {\left( {{\text{mm}}/{\text{min}}} \right)}}}\right.\kern-\nulldelimiterspace} \!\lower0.7ex\hbox{${\left( {{\text{mm}}/{\text{min}}} \right)}$}}$$, $$K_{p} = 37626{\raise0.7ex\hbox{${{\text{Ns}}}$} \!\mathord{\left/ {\vphantom {{{\text{Ns}}} {\text{m}}}}\right.\kern-\nulldelimiterspace} \!\lower0.7ex\hbox{${\text{m}}$}}$$, $$K_{{{\text{pi}}}} = 29.3{\raise0.7ex\hbox{${\text{V}}$} \!\mathord{\left/ {\vphantom {{\text{V}} {\text{A}}}}\right.\kern-\nulldelimiterspace} \!\lower0.7ex\hbox{${\text{A}}$}}$$, $$T_{n} = 7.3\,{\text{ms}}$$, $$T_{g} = 0.246\,{\text{ms}}$$, $$K_{a} = 0.9975$$. The comparison of angle simulation results is shown in Fig. 6. The above figure is the angle change of the reference, feedback, and NX measured curves, the middle figure is the deviation of reference and feedback curve, and the figure below is the deviation of reference and NX measured curve.

### Co-simulation results

**Fig. 7:**
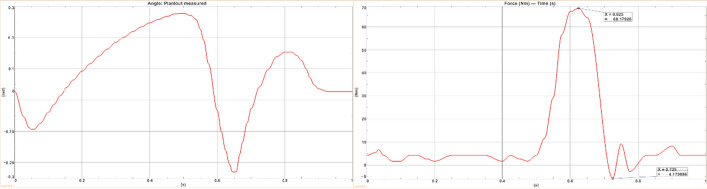
Ankle joint characteristic curve obtained by co-simulation in NX

The angle and torque result curves extracted in NX are shown in Fig. 7. It can be seen from the Fig. 7 that the range of angle and torque of the ankle joint in the co-simulation results of NX and Simulink is basically the same as the range of normal human ankle joint motion obtained in the experiment. The angle change trend conforms to the angle change trend during a complete gait cycle. The obtained trajectory tracking curve and the displacement data obtained from the experiment can achieve high-precision control, and the error can be controlled within $- 2.5 \times 10^{ - 2} \sim 2.5 \times 10^{ - 2} \,{\text{mm}}$. This value is less than the human sensory threshold, which proves the feasibility of active and passive ankle joint prosthesis.

During the simulation process, the prosthetic model structure is used as a rigid body for calculation, use Signal Builder to collect points as the input source of the angle reference signal, and there is a certain sampling interval and slightly different sampling frequency between Simulink and NX, which will cause certain errors, such as signal jitter and fluctuation. Therefore, there is a certain difference between the simulation curve and the gait experiment results, but overall, there is a higher close rate, which reflects the real time and effectiveness of the artificial limb joint impedance control algorithm. After the prosthetic joint adopts parallel springs, it has passive characteristics similar to biological joints, reducing part of the mechanical energy required by the motor. The actual peak torque required by the motor is 6 8Nm, which is less than the peak torque of human joints of 75 Nm. The prosthesis can meet the high instantaneous power (about 250 W) required for walking under lower power-driven conditions (for example, 150 W. in this study), and improve the energy efficiency of the prosthetic system. In the study, the joint rotation angle curve was used to simulate the ground restraint change, which will cause the prosthetic driver to compress the parallel spring in the swing phase and output additional mechanical energy. In actual control, the trajectory control of the joint angle in the swing phase can be further optimized to achieve precise control.

## Discussion

Based on the principle of tandem spring driver, this paper promoted an active and passive ankle prosthesis suitable for lower limb amputation. This article mainly carried out the following research work:

1. Based on the human ankle joint anatomy and gait experiments, the biomechanical characteristics of the ankle joint are discussed, the requirements and parameters of the active and passive ankle joint prosthesis are determined. The motion characteristics are analyzed, and it is determined that the prosthesis has two degrees of freedom on the sagittal and coronal planes, which realized the active gait cycle of sagittal plane plantar/dorsiflexion movement and the passive movement of coronal plane inversion/eversion.

2. According to the parameters, requirements and movement characteristics of the prosthesis, the active and passive ankle joint prosthetic mechanism are based on the principle of the SEA. Including the determination of the motor drive as the driving force source of the series spring driver; the SEA transmission module and elastic element are designed; the component construction and overall assembly of the active and passive ankle joint prosthesis are completed based on CATIA.

Referred to the power ankle joint prosthesis developed by the Herr team of MIT [12–14]. SEA is a series combination of a motor and a spring mechanism, which has a certain degree of flexibility. At the same time, Wang X. S.'s team [15] added parallel springs in addition to the SEA to form an active and passive driving mode. In addition, in the lower limb prosthesis designed by Vallery of the University of Michigan [16], there is a clutch between the spring at the ankle joint and the foot plate, and the motor drives the clutch switch. The spring is continuously compressed in the early stage of the support, and the clutch is locked when the compression reaches the limit, the clutch is opened when the toe pressure signal is generated, and the released spring energy pushes the human body forward. Bergelin of Marquette University [17] designed a power ankle joint prosthesis. The ankle joint torque is the sum of the initial spring torque, the spring torque and the motor torque. Cherelle of the University of Brussels [18] developed AMP-Foot 2.0, which uses a low-power electric motor to store energy in a spring and release it at an appropriate time, which can effectively utilize the energy, but cannot precisely control the angle of the ankle joint. Wang Q. N. team of Peking University [19] has developed a PANTOE with rigid adaptable ankle and toe joints, driven by tandem elastic actuators. Experiments have shown that the ankle and toe angles of the prosthesis are close to those of healthy limbs.

At present, most laboratories research on powered ankle joint prostheses are at the stage of experimental prototypes, and pay less attention to quality, volume, and energy consumption. As a driving source, motors generally have conflicts between output torque, motor size and cost. Therefore, in the clinical application of dynamic ankle joint prostheses, attention should be paid to the optimization of the mechanism, so that the mechanism can be integrated, lightened and reflected flexibility and adaptability to a greater extent under the condition of meeting performance.

3. A compound P/PI feedback control system is designed according to the motion characteristics of the ankle joint. Using NX Nastran and Matlab/Simulink modules to co-simulate the system, through the design of the control system, the kinematics performance of the prosthetic mechanism is analyzed. It is verified that the system can meet the control requirements of the active and passive ankle joint prosthesis system and the feasibility of this kind of prosthesis.

The Herr team of MIT first proposed FSM control [12]. The support period is divided into three states: controllable plantar flexion, controllable dorsiflexion and dynamic plantar flexion. Angle and pressure sensors detect the behavior of the amputee to determine the walking state. During the state, the motor generates corresponding impedance to control the plantar/dorsiflexion of the ankle joint prosthesis. The foot and ankle prosthesis developed by the Herr team and the electric knee–ankle prosthesis developed by the Goldfarb team at Vanderbilt University [20] also use this control method. The latter proposes a finite-state adaptive controller based on this method. Actively adjust the relationship between the residual limb and the prosthetic foot during the support period to reduce the sagittal moment transferred to the residual limb [21]. Cao H. team of ECUST [22] judges the gait cycle of the prosthesis according to the FSM, and selects the required torque or angle of the corresponding control law output. In the later research work, referring to the research of Cao H.'s team [23], the feedback compensation force inner loop was added to the impedance control algorithm, which improved the controllability of the active and passive hybrid drive ankle joint prosthesis, and improved the control performance of the joint impedance. Ficanha et al. [24] designed an ankle joint prosthesis. The FSM can switch between the impedance/ admittance controllers based on the gait. Compared with the prosthesis using the position controller, this method can reduce the torque required at the ankle joint and realize the right Tracking of reference trajectories.

Due to individual differences in gait, the static parameters of each state of the FSM usually need to be optimized and adjusted through complex training [25]. The FSM control divides the pre-designed gait trajectory into sections, and designs the control law for each stage separately, which is relatively simple to implement. The processor has a small amount of calculation and high practicability, but it will cause the prosthetic gait to be incoherent. If too many control parameters are used, the process of adjusting the parameters will become difficult, and real-time adjustment cannot be achieved. Once disturbances occur in the system, the control effect will deteriorate [26].

## Conclusion

Based on the motion characteristics of human ankle joint prostheses, this paper completes an effective and feasible design of active and passive ankle joint prostheses. Based on the design of active modules, passive modules and bionic joints, the overall weight of the prosthesis is greatly reduced; the efficient transmission unit reduces power loss, makes the design of the prosthetic structure more streamlined. This article preliminarily proposes an active and passive hybrid prosthesis, which coordinates the advantages of active and passive control to meet the needs of the human body and solve some of the shortcomings of the current dynamic ankle joint prosthesis. Meanwhile, the compound P/PI feedback control system is used to verify the feasibility of the design scheme. Because the FSM control is widely used, mature, simple to implement, and the processor has a small amount of calculation, it can better realize the biological characteristics of the human ankle joint. The active and passive ankle joint prosthesis development process and prosthetic design requirements used in this study have a certain reference value and provide a certain reference for the promotion of lower limb prosthetic products.

In the subsequent research, the following aspects can be continued to improve:

1. Non-metallic materials can be considered for the optimization of the mechanical structure of the prosthesis and the reduction of the overall weight. The smaller weight means that the energy consumption of the prosthesis is reduced, which is of great benefit to enhancing the endurance of the prosthesis.
2. Afterwards, make prototypes, carry out relevant experimental verification, and then optimize and calibrate the prototype design based on the test results.
3. The stiffness of the human ankle joint is variable, and the stiffness of the tandem spring driver is constant. To achieve the bionic performance of the prosthesis, the design of the driver should be further explored to make it have variable stiffness characteristics.
4. The research on the biomechanical properties of lower limbs is based on the gait experimental data of healthy people. There is no comparison item. The experiment should be improved to collect lower limb kinematics data of people with lower limb disabilities.
5. Direct volition control is based on the EMG signal, and the wearer has a high degree of autonomy in walking, so that the combination of EMG signal and deep learning will become a hot spot and trend in the future. Because pure biomechanical signal control and bionic signal control have their own characteristics. It can be considered to combine the two types of signals to achieve inclusiveness and improve the control effect and accuracy.

## Methods

**Fig. 8:**
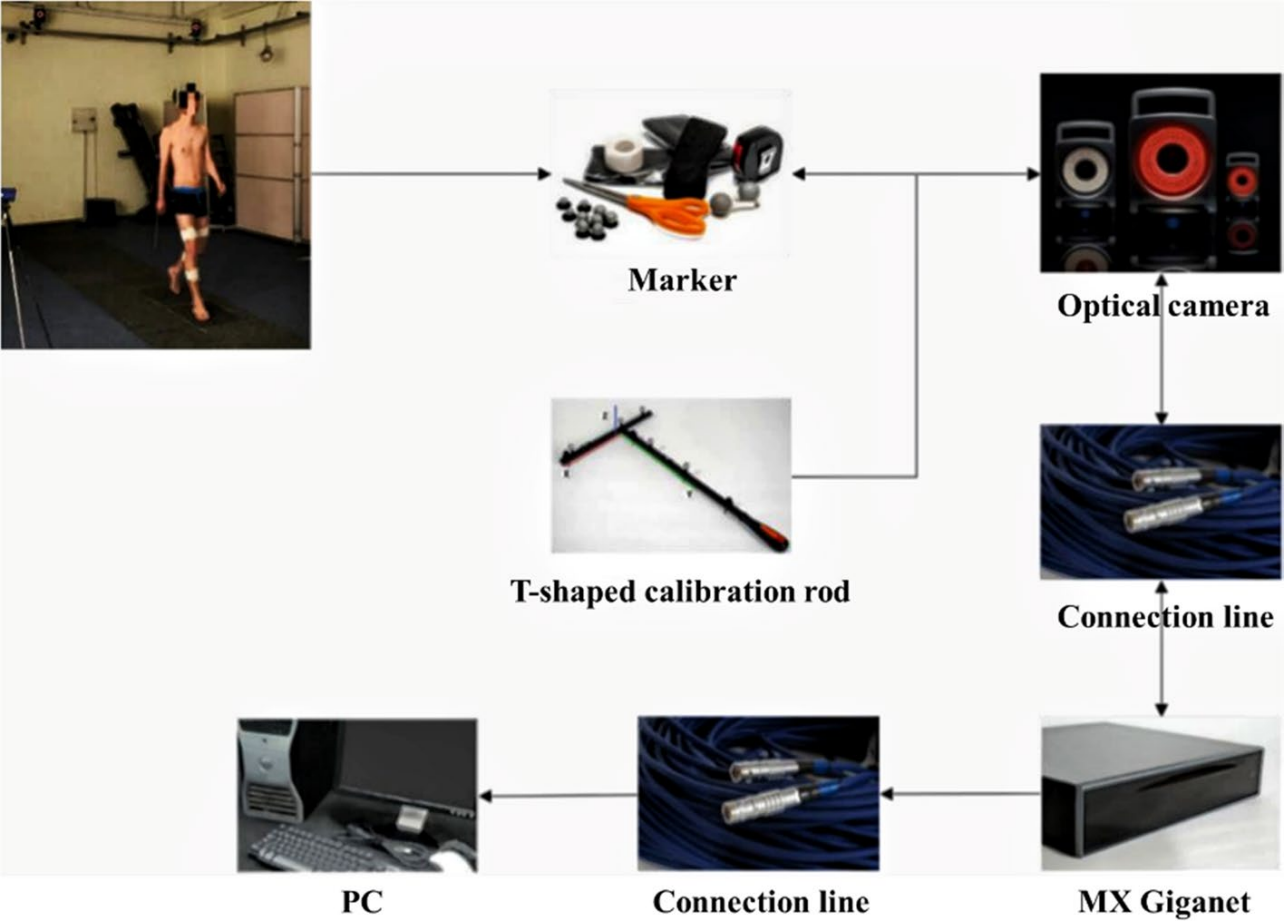
Principle of motion capture system

Vicon motion capture system is a passive optical motion capture system, which is widely used in medical fields, such as biomechanical experiments, gait analysis, spinal motion range and spinal stability evaluation. Research has found that the motion capture system has guiding significance for the design and production of prosthesis. The system can collect human motion parameters in the prosthetic design process [27]. The main components of the Vicon motion capture system include optical cameras, MX Giganet, PC, reflective markers, etc. The working principal diagram is shown in Fig. 8.

Paste the Marker to the specific position of the target to be measured. When the red light emitted by the optical camera shines on the surface of the Marker, the Marker will reflect the same wavelength of light, then the camera will determine the 2D coordinates of the Marker and shoot continuous pictures. The data collected by the optical camera will be stored in the MX Giganet, and then transmitted to the PC through the cable, and processed by the PC control software to obtain the 3D coordinates of the Marker. The high-speed, high-resolution camera can clearly capture the continuous motion trajectory of the Marker fixed on the target to be measured, and obtain the final motion data after preprocessing.

### Gait experiment

**Fig. 9:**
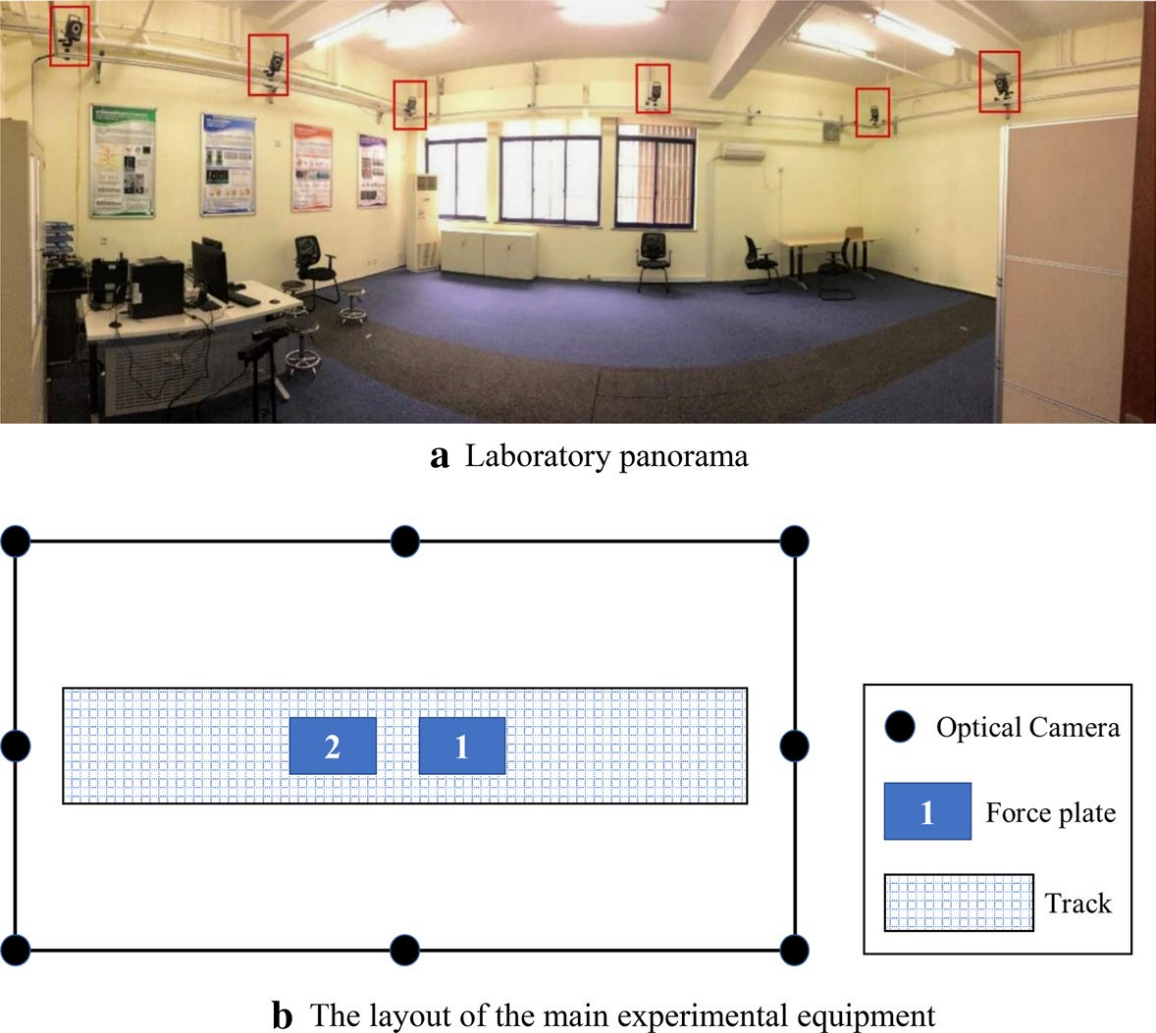
Layout of laboratory facilities

**Fig. 10:**
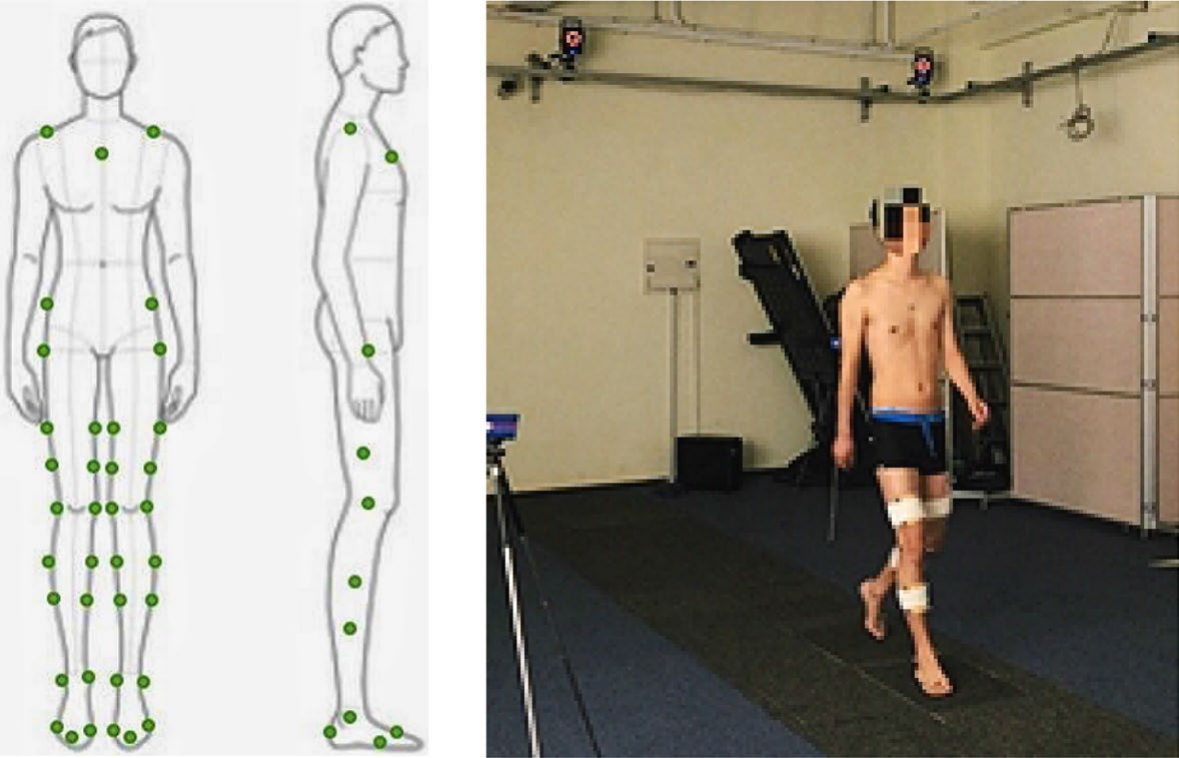
Markers' fixed position

The experiment collected data related to human gait when walking on flat ground. To avoid external light from interfering with the reflective markers, the experiment was carried out on flat ground in a dark room. A total of 8 optical cameras (VICON T40) are used in the experiment, and there are 2 force plates in the middle of the laboratory, 508  mm length, 464 mm width, the experimental site is about 10 m long, 5 m wide, and 3 m high, cameras are distributed around the site. The site layout is shown in Fig. 9. The Vicon motion capture system is achieved by capturing the reflective markers fixed on the tester, so the location of the markers is very important. As shown in Fig. 10, the reflective markers are, respectively, fixed on the target's shoulders, chest and waist and hips, and are symmetrically distributed on the lower limb joints on both sides, the outer thigh, the outer calf, and the heel.

The study was approved by the Ethics Committee of Shanghai Jiao Tong University Affiliated Sixth People’s Hospital (No. 2020-15). Inclusion criteria: 20 male volunteers aged 20 ~ 24 years, 170 ~ 185 cm tall, 65 ~ 78 kg weighing, in good physical condition and no clear history of trauma (high energy or low energy). Exclusion criteria: younger than 20 years or older than 25 years, female, imaging examination of the affected or healthy side of the hip joint structure abnormalities, such as flat hip, femoral head and neck dysplasia, or pathological fracture of the femoral neck, hip osteoarthritis or have internal plants. Each tester needs to measure height, weight, leg length, knee width and ankle width before performing the gait experiment. At first, perform static capture. The tester stands on a force plate and maintains a static standing posture to establish an initial rigid body model. Second, perform real-time dynamic capture. The tester walks back and forth in the test area at the usual walking speed. It should be noted that each foot of the tester should step on a force plate during walking; meanwhile, the arm cannot block the reflective markers when it is swinging, and each group of experiments was performed three times. When the tester moves in the collection area, the movement of the rigid body model matching the marker can be seen in the Nexus interface, the collected data can be viewed in the database, and the data is finally saved in scv format for output.

### Data analysis

**Fig. 11:**
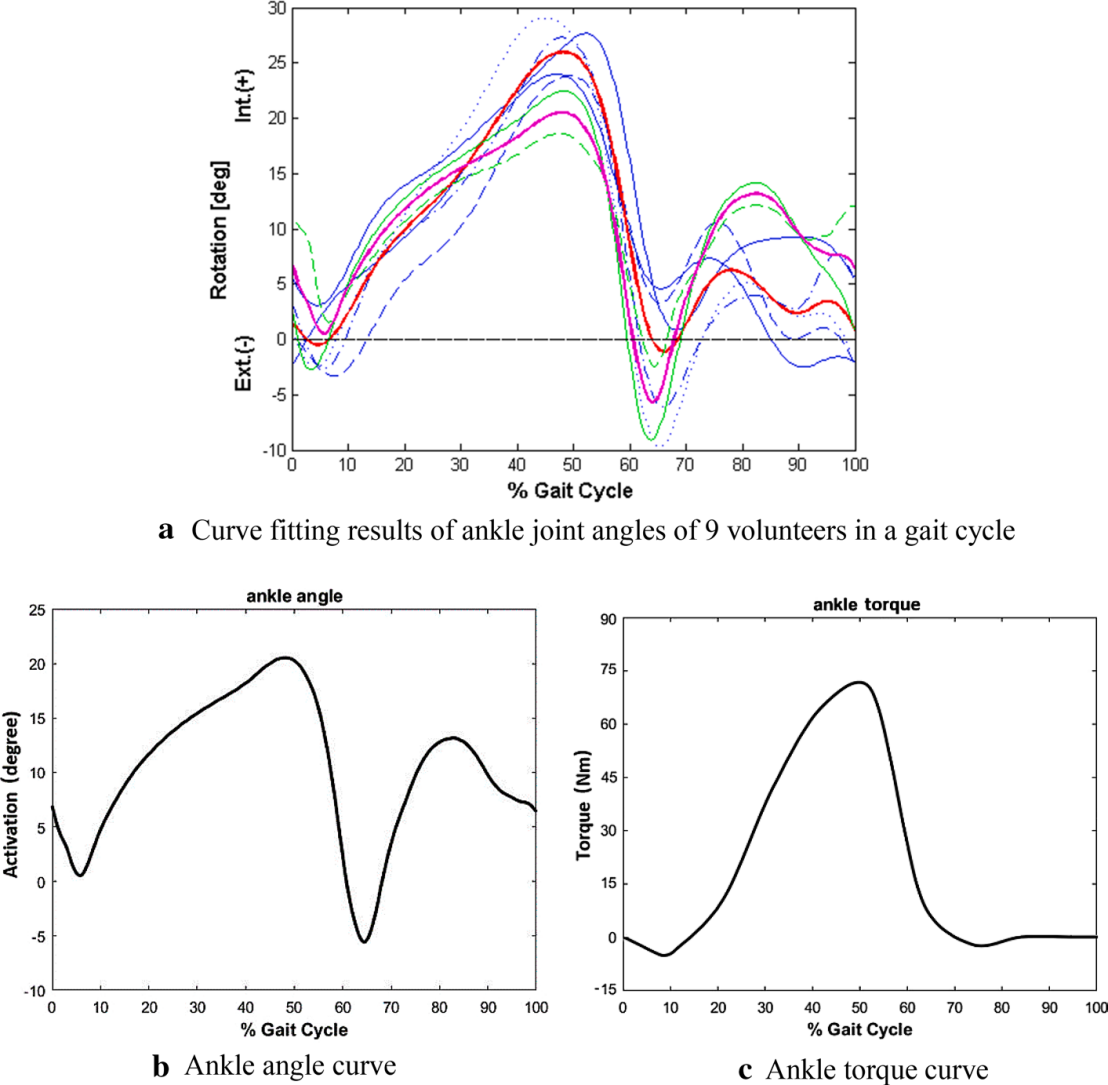
Characteristic curve of ankle joint

The optical camera captures the various motion parameters of the tester during the movement by capturing the reflective markers. This part of the data is preprocessed and exported by Vicon Nexus. A complete gait cycle is intercepted in each set of data. Due to differences in the stride length and pace of different testers, the time and data volume corresponding to a gait cycle collected are also different. Therefore, time cannot be used as a unified standard when analyzing. Use Matlab to process the gait cycle data. First, a gait cycle is divided into 100 parts equally, that is, the data whose abscissa is time is equivalently replaced with a gait cycle, so that the abscissa has the same numerical interval. The fitting results of ankle angle curve of 9 volunteers in one gait cycle are shown in Fig. 11a. Second, calculate the average value of the angle and torque of each movement curve of 20 testers. Therefore, in the complete gait cycle range, the newly obtained ankle joint angle and torque curve is shown in Fig. 11b, c.

**Fig. 12:**
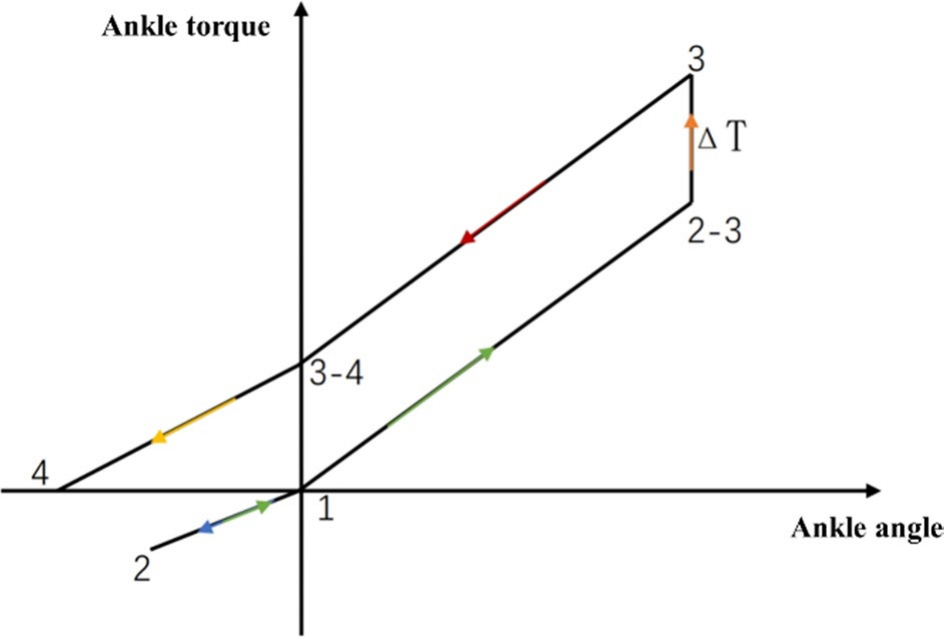
Ankle joint Angle—Torque ideal line chart

According to the gait cycle phase analysis and ankle joint characteristic curve of the human body during normal walking, the characteristic curve can be ideally converted into a broken line, as shown in Fig. 12. The 1 → 2 stage is the stage of plantar ground contact cushioning and energy storage, has linear characteristics, so linear series springs can be used as energy storage; The 2 → 3 stage is non-linear, so series springs and parallel springs can be used together. Point 3 is the maximum ankle joint torque, and the difference compensation torque from point 2–3 to point 3 can be provided by the motor; The 3 → 4 stage is to release energy, and at the same time, it is necessary to provide additional energy to push the human body forward on the ground.

### Design of ankle joint prosthesis

After the motion analysis of a gait cycle is measured, a three-dimensional model of the active and passive ankle joint prosthesis needs to be established. Regarding the selection of the driving force source, considering the power requirements, compact structure and lightweight requirements of the prosthesis, it is more reasonable to comprehensively consider the use of motor drive. The structural design refers to the dynamic ankle joint prosthetic promoted by the team of Professor Hugh Herr MIT [12–14]. The motor is used as the driving force source to drive a linear ball screw. Through the rotation of the screw shaft, the nut slides linearly along the axis of the screw. At this time, the rotary motion output by the motor is transformed into linear motion. The output end of the screw nut is connected in series with an elastic element to form a complete structure of the SEA. The elastic element is connected with the plantar part of the prosthesis to form a complete power transmission device. Under the action of the linear sliding of the nut, the ankle joint prosthesis has plantar flexion and dorsiflexion angles. At the same time, a parallel elastic element is arranged at the front end of the foot and plantar to limit the dorsiflexion angle of the ankle joint prosthesis. The design parameters of active and passive ankle joint prosthesis are shown in Table 2.

**Table 2:** Design parameters of ankle prosthesis

Parameter	Value
Ankle range of motion /$(^\circ )$	− 5 ~ 20
Ankle Angular Velocity / (rad/s)	0.5 ~ 1.5
Maximum torque of ankle joint / (N·m)	75

**Fig. 13:**
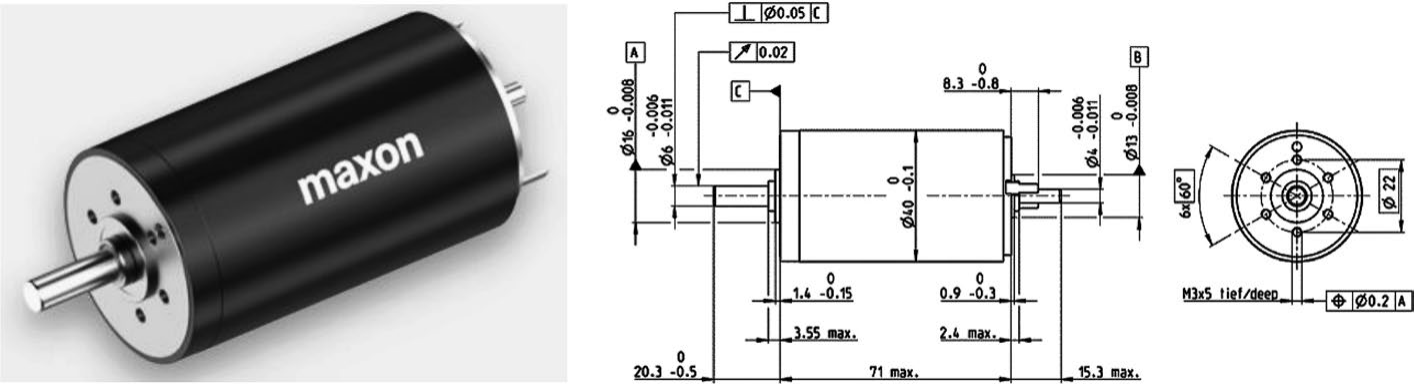
R40 appearance and dimensions

As the core driving force source of the ankle joint prosthesis, the electric motor is very important to whether the series elastic actuator can play a role. The selection of the motor requires that it can output a larger speed and torque under a lower weight to meet the requirements of lightweight and high driving force of the prosthesis. Upon request, the information found that the Swiss Maxon brand motors have high adaptability. According to the use scene conditions of the prosthesis, the type of motor is determined: DC motor. The DC motor can withstand frequent impact loads, can continuously output rated torque at low speeds, and can achieve frequent rapid start, braking and reverse rotation. Select the DC servo motor of model RE40. The appearance and relevant characteristic parameters of the motor are shown in Fig. 13 and Table 3.

**Table 3:** Maxon RE40 characteristic parameters

Parameter	Value	Parameter	Value
Rated Voltage	$U_{0} = 48 \,{\text{V}}$	Locked-rotor Current	$I_{\max } = 42.2 {\text{A}}$
Rated Speed	$$n_{0} = 7000\,{\text{ rpm}}$$	Speed Constant	$$W_{n} = 158 {\raise0.7ex\hbox{${{\text{rpm}}}$} \!\mathord{\left/ {\vphantom {{{\text{rpm}}} {\text{V}}}}\right.\kern-\nulldelimiterspace} \!\lower0.7ex\hbox{${\text{V}}$}}$$
Rated Power	$P_{0} = 150\, {\text{W}}$	Idling Speed	$n_{1} = 7590\,{\text{rpm}}$
Locked-rotor Torque	$T_{\max } = 2560\,{\text{ mN}} \cdot m$	Moment of Inertia	$J = 137 {\text{g}} \cdot \le {\text{cm}}^{2}$
Rated Torque	$T_{0} = 187 \,{\text{mN}} \cdot \le m$	Weight	$m = 480 g$
Torque Constant	$$T_{n} = 60.3\, {\raise0.7ex\hbox{${{\text{mN}} \cdot \le {\text{m}}}$} \!\mathord{\left/ {\vphantom {{{\text{mN}} \cdot \le {\text{m}}} {\text{A}}}}\right.\kern-\nulldelimiterspace} \!\lower0.7ex\hbox{${\text{A}}$}}$$	Effectiveness	$$\eta = 0.92$$

The rated voltage of the motor $U_{0} = 48V$, to ensure the safety of the wearer, the maximum working voltage provided by the motor is $36V$, and the maximum working speed $n_{max}$ of the motor is: $n_{{{\text{max}}}} = U_{1} \times W_{n} = 36 \times 158 = 5688\,{\text{rpm}}$. The torque provided by the motor is proportional to the current of the motor, $M_{m}^{\max } = T_{n} I_{\max } \frac{{U_{1} }}{{U_{0} }}$. Motor torque constant $T_{n} = 60.3mNm/A$, the working voltage of the motor is set to $36V$, so the maximum torque that the motor can provide is $M_{m}^{\max } = 1.92\,{\text{Nm}}$.

### Structure of the ball screw

**Fig. 14:**
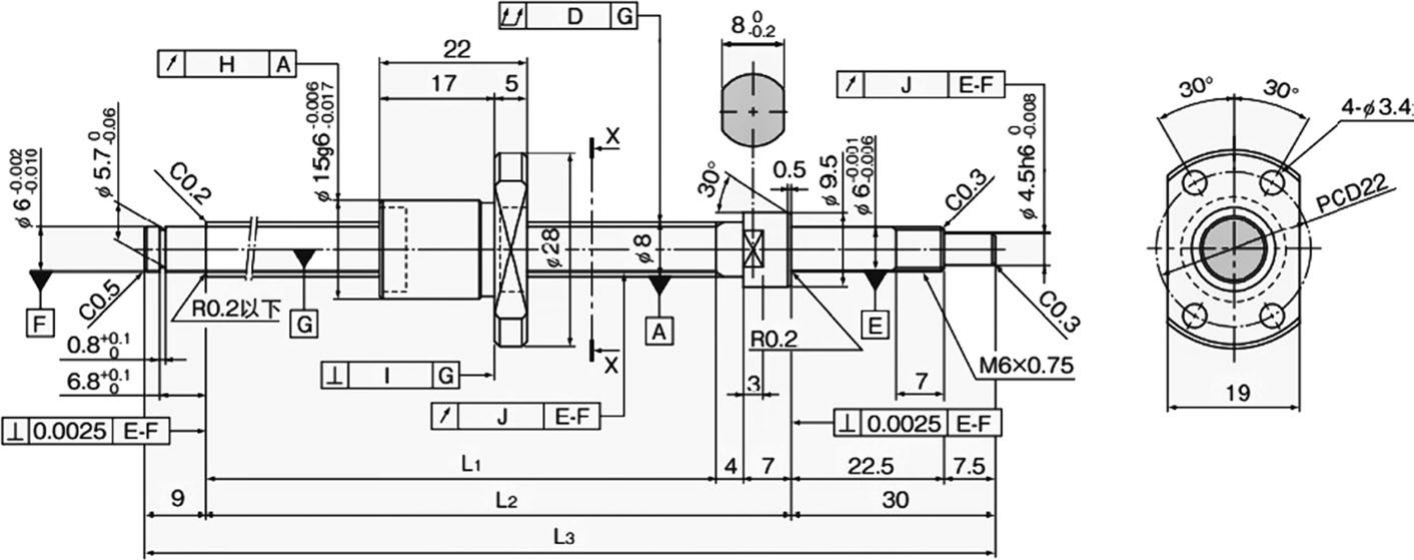
The structure of the ball screw

The ball screw is the main component of the transmission unit of the series elastic drive, and its output torque directly acts on the executive end, that is, it drives the motion of the ankle joint prosthesis. Therefore, the output torque must meet certain requirements to meet the motion requirements of the ankle joint prosthesis. Through the general selection method, finally selected a ball screw with a shaft diameter of 8 mm, a lead of 2 mm, a total length of 125 mm, and a stroke of 40 mm. The relevant size specifications are shown in Fig. 14 and Table 4. Through the gait experiment, the maximum torque required by a person during normal walking is $75{\text{N}} \cdot {\text{m}}$, and the peak appears at the beginning of the dynamic plantar flexion movement in the gait cycle. The maximum torque provided by the motor $M_{m}^{\max } = 1.92\,{\text{Nm}}$. After the torque is amplified by the transmission mechanism, the relationship between the tensile force $F_{BS}$ output by the ball screw on the actuator and the maximum torque of the motor is as follows: $F_{{{\text{BS}}}} = \frac{{2\pi M_{m}^{\max } }}{{RP_{h} }}, P_{h} = 2\,{\text{mm}}, R = 0.92$. The maximum tensile force output by the ball screw is calculated as $F_{{{\text{BS}}}} = 6556\,{\text{N}}$. The tensile force output by the screw directly acts on the actuator and drives the prosthetic foot structure to rotate around the bionic ankle joint. Therefore, the maximum torque that the tensile force transmitted by the screw can provide is: $T_{{{\text{ank}}}} = F_{{{\text{BS}}}} r_{f}$, $r_{f}$ is the distance between the ball screw and the center of rotation of the ankle joint, which is 20 mm. Therefore, the maximum torque that the driver can provide is $T_{{{\text{ank}}}} = 131\,{\text{Nm}}$, which is greater than the maximum torque required by the human body obtained from the experiment, so the torque provided by the motor meets the design requirements.

**Table 4:** Dimensional parameters of ball screw

Parameter	Value	Parameter	Value
Lead	$P_{h} = 2\,{\text{ mm}}$	Weight	$m = 68 {\text{g}}$
Shaft diameter	$D = 8 \,{\text{mm}}$	Stroke	${\text{ST}} = 40 \,{\text{mm}}$
Length	$L_{1} = 75\,{\text{ mm}}$	Number of Turns	$1 {\text{Circle}} \times 3 {\text{Row}}$
	$L_{2} = 86 \,{\text{mm}}$	Basic Static Load Rating	$C_{oa} = 2.3 {\text{kN}}$
Full Length	$L_{3} = 125\,{\text{ mm}}$	Basic Dynamic Load Rating	$C_{a} = 1.4 {\text{kN}}$

### Design of elastic structure

**Fig. 15:**
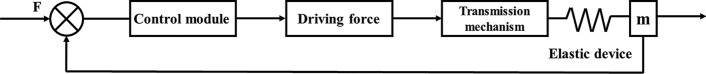
Structure diagram of series spring driver

To better realize the bionic performance of the ankle joint prosthesis, the SEA with compliant characteristics was selected. The structure can produce a certain deformation when a collision occurs in the environment, play a role in buffering and shock absorption, and has the characteristics of good energy storage, impact resistance, etc., which has been widely used in the field of walking robots. The typical structure of the SEA is shown in Fig. 15, which is composed of a control module, a drive module, a transmission module, an elastic element and an end effector (load) in series. Its working principle is to directly or indirectly connect an elastic device in series through a driving force source, output a flexible force, and then act on the end force object [28, 29]. A customized metal structure spring has been chosen in this paper. The design process of this kinds of spring is generally determined by the designer according to actual needs to determine the target parameters, such as the structure, size, and stiffness coefficient of the elastic element, to determine the initial design of the spring. In the active and passive ankle joint prosthesis in this article, the elastic element as a SEA is the key to achieving the flexibility of the prosthesis, and the rotational stiffness of the elastic element should be paid attention. During the process of the series spring, an *L*-shaped spring was designed by deforming on the basis of the leaf spring. The input end is connected with the ball screw, and the output end is connected with the structure of the prosthetic foot plantar part.

The parallel spring is located at the front end of the ankle joint prosthesis. When the plantar/dorsiflexion angle of the prosthesis reaches a certain angle during the movement, the parallel spring will be stretched or compressed to generate additional pressure, provide an additional ankle joint torque. At the same time, it also plays a certain restrictive role to prevent the rotation angle of the prosthetic ankle joint from being too large. Parallel springs can store the gravitational potential energy when the center of gravity drops during the controlled dorsiflexion phase, and at the same time release energy to provide additional torque during the dynamic plantarflexion phase. Therefore, it is convenient to choose a smaller size motor and reduce the overall weight of the prosthesis.

In order for the parallel spring to output enough torque in the later stage of support, the choice of elastic stiffness is particularly important. The rotational stiffness of the parallel spring is set to $500\,{\text{Nm}}/{\text{rad}}$. When the ankle joint moves to the maximum dorsiflexion angle $\theta_{D}^{\max } = 20^\circ \left( {0.35\,{\text{rad}}} \right)$, the maximum torque that the parallel spring can provide $M_{P}^{\max }$:

$$M_{P}^{\max } = K_{P} \theta_{D}^{\max } = 175\,{\text{Nm}}{.}$$

Energy storage relationship of parallel springs: $W_{P} = \frac{1}{2}K_{P} \left( {\theta_{D}^{\max } } \right)^{2}$.

It is calculated that the stored energy of the parallel spring is $30.6J$, which meets the needs of human movement.

Through the analysis of the biomechanics of the ankle joint, it can be seen that the angle of movement of the ankle joint in the sagittal plane is $25^\circ \sim 30^\circ$ downward plantar flexion and upward $15^\circ \sim 20^\circ$ dorsiflexion, and the variation range of the inversion angle of the coronal plane is $5^\circ \sim 10^\circ$.. The movement of a normal joint has a certain range of angle changes, which is related to the changes in the tension produced by the man body’s own ligaments. The ligaments play a role in ensuring the stability of the joint when the ankle joint moves. When designing the prosthesis structure, it can be similarly designed as a prosthetic movement angle limiting device.

**Fig. 16:**
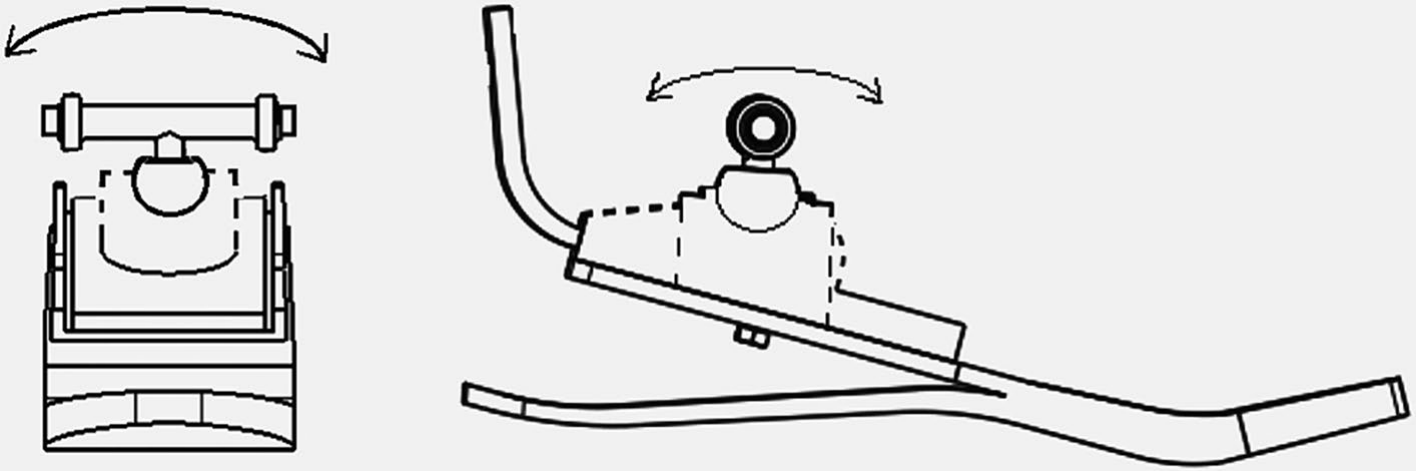
Ankle joint prosthesis structure diagram

The human ankle joint has 3 degrees of freedom of rotation on the plane, of which dorsiflexion and plantar flexion in the sagittal plane are the most important and must be possessed by ankle joint prostheses. The ankle joint prosthesis designed in this paper has two degrees of freedom of rotation on the plane, and a limit device is added to the horizontal plane of the spherical ankle joint to restrict the rotation on the horizontal plane. The design of the ankle joint structure of the prosthesis is shown in Fig. 16.

The plantar/dorsiflexion movement of the ankle joint prosthesis designed in this paper is active movement in the sagittal plane. The coronal valgus/inversion movement is passive movement. Passive movement will only occur when the prosthesis is subjected to lateral forces. For example, when walking on uneven roads, the prosthesis will turn in or out when it comes into contact with the ground, and it will return to the original state when the prosthesis is off the ground. The angle variation range on the coronal plane is $-10^\circ \sim 10^\circ$. The design of the spherical ankle joint makes the movement of the prosthesis more flexible and greatly improves the comfort of the prosthesis wearer. The simple structure design makes the overall structure of the prosthesis more compact and reduces the weight of the prosthesis.

### Construction of control algorithm

**Fig. 17:**
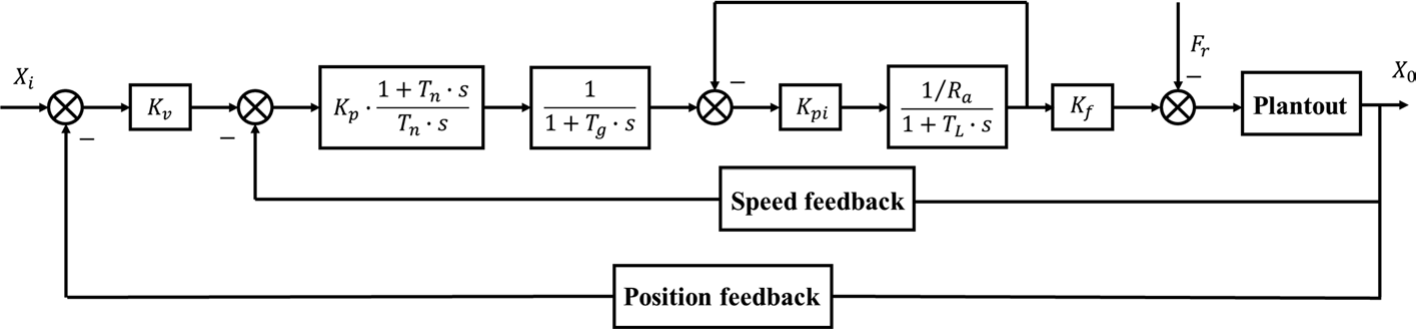
Angle control system transfer function block diagram

The basic structure of the control system of the active and passive ankle joint prosthesis studied in this paper is the same as that of the general closed-loop control system. It adopts a fully closed-loop control with the position loop as the outer loop, the speed loop and current loop as the inner loop. The principle of the control structure is shown in Fig. 17. $X_{i}$ is the input value of the position, $X_{0}$ is the output value of the position, $K_{v}$ is the position loop proportional gain, $K_{p}$ is the speed loop proportional gain, $T_{n}$ is the speed loop integral response time constant, $T_{g}$ is the speed loop filter time constant, $K_{pi}$ is the current loop proportional gain, $R_{a}$ is the motor armature resistance, $L_{a}$ is the motor armature inductance, and $K_{f}$ is the force constant [8].

Among them, the proportional gain of the current loop can improve the response speed of the controller; The speed loop proportional integral link can increase the speed loop response speed and improve the stability of the speed loop; The speed filter can filter out the high-frequency interference signal output by the speed loop, improve the stability of the speed signal, reduce the interference caused by the speed loop output signal to the current loop input, and reduce the negative influence caused by its direct action; The proportional gain of the position loop can enhance the response speed of the entire system and improve the positioning accuracy of the servo system.

The position command value sent by the control system passes through the position loop to get the speed command value. Then the PI regulator of the speed feedback loop reduces the steady-state error. The high-frequency interference signal is filtered out through the speed filter link to enhance the stability of the speed. After that, the current loop adjusts the actual output value of the current. Finally, the force regulator is used to control the output torque of the rotating motor, and drive the movement of the prosthesis, and obtain the transfer function of the control structure of the entire system [9]:

$$G_{h} \left( s \right) = \frac{{K_{p} K_{pi} K_{a} K_{f} \left( {1 + T_{n} s} \right)}}{{T_{n} s\left( {1 + T_{g} s} \right)\left( {T_{L} s + K_{pi} K_{a} + 1} \right)}}$$

Among them:$$K_{a} = \frac{1}{{R_{a} }}$$, $$T_{L} = \frac{{L_{a} }}{{R_{a} }}$$.

## Data Availability

The data sets used and/or analyzed during the current study are available from the corresponding author on reasonable request.

## Abbreviations

CAD/CAE/CAM: Computer Aided Design/Computer Aided Engineering/Computer Aided Manufacturing
P/PI Controller: Proportional/ Proportional Integral Controller
EMG: Electromyogram
PC: Personal Computer
MIT: Massachusetts Institute of Technology
JLU: Jilin University
ECUST: East China University of Science and Technology
UCAS: University of Chinese Academy of Sciences
SEA: Series Elastic Actuator
FSM: Finite State Machine
